# Mapping Thematic Trends and Analysing Hotspots Concerning the Use of Stem Cells for Cartilage Regeneration: A Bibliometric Analysis From 2010 to 2020

**DOI:** 10.3389/fphar.2021.737939

**Published:** 2022-01-03

**Authors:** Demeng Xia, Jianghong Wu, Feng Zhou, Sheng Wang, Zhentao Zhang, Panyu Zhou, Shuogui Xu

**Affiliations:** ^1^ Department of Orthopedics, Naval Hospital of Eastern Theater, Zhoushan, China; ^2^ Department of Orthopedics, Changhai Hospital, Naval Medical University, Shanghai, China; ^3^ Department of Emergency, Affiliated Hospital of Jiangsu University, Jiangsu, China; ^4^ Department of Orthopedics, Naval Medical University, Shanghai, China

**Keywords:** stem cells, cartilage regeneration, bibliometric analysis, hotspot, web of science

## Abstract

**Background:** Defects of articular cartilage represent a common condition that usually progresses to osteoarthritis with pain and dysfunction of the joint. Current treatment strategies have yielded limited success in these patients. Stem cells are emerging as a promising option for cartilage regeneration. We aim to summarize the developmental history of stem cells for cartilage regeneration and to analyse the relevant trends and hotspots.

**Methods:** We screened all relevant literature on stem cells for cartilage regeneration from Web of Science during 2010–2020 and analysed the research trends in this field by VOSviewer and CiteSpace. We also summarized previous clinical trials.

**Results:** We screened 1,011 publications. China contributed the largest number of publications (317, 31.36%) and citations (81,376, 48.61%). The United States achieved the highest H-index (39). Shanghai Jiao Tong University had the largest number of publications (34) among all full-time institutions. The Journal of *Biomaterials* and *Stem Cell Research and Therapy* published the largest number of studies on stem cells for cartilage regeneration (35). SEKIYA I and YANG F published the majority of articles in this field (14), while TOH WS was cited most frequently (740). Regarding clinical research on stem cells for cartilage regeneration, the keyword “double-blind” emerged in recent years, with an average year of 2018.75. In tissue engineering, the keyword “3D printing” appeared latest, with an average year of 2019.625. In biological studies, the key word “extracellular vesicles” appeared latest, with an average year of 2018.9091. The current research trend indicates that basic research is gradually transforming to tissue engineering. Clinical trials have confirmed the safety and feasibility of stem cells for cartilage regeneration.

**Conclusion:** Multiple scientific methods were employed to reveal productivity, collaborations, and research hotspots related to the use of stem cells for cartilage regeneration. 3D printing, extracellular vesicles, and double-blind clinical trials are research hotspots and are likely to be promising in the near future. Further studies are needed for to improve our understanding of this field, and clinical trials with larger sample sizes and longer follow-up periods are needed for clinical transformation.

## 1 Introduction

Articular cartilage (AC) is a layer of hyaline cartilage with unique viscoelastic characteristics. Its principal function is to provide a smooth, lubricated surface for articulation and to facilitate the transmission of loads with a low frictional coefficient which disperses the stress in the joint to protect it from defects (Sophia Fox et al., 2009). However, traumatic and degenerative injuries of AC and untreated cartilage injuries eventually progress to osteoarthritis (OA), which is the most common type of musculoskeletal disorder (Carballo et al., 2017). Articular cartilage has very poor healing capability; because of its avascular nature, mature chondrocytes have a weak ability to proliferate and produce sufficient extracellular matrix (ECM) to fill a defect (Le et al., 2020). A large number of studies have been carried out to simulate cartilage regeneration. Surgical techniques to treat focal chondral defects include marrow stimulation (MST) surgery, such as the current first-line treatment microfracture (MF) surgery. Although the resulting ‘fibrocartilage’ provides some symptomatic relief, it has substantially weakened mechanical properties compared with those of normal articular cartilage (Toh et al., 2010; Murphy et al., 2020). In comparison, cell-based regenerative therapy can provide a long-term solution, alleviate symptoms and ultimately delay OA progression. Autologous chondrocyte implantation (ACI) has been approved by the FDA and is currently in clinical practice. ACI requires isolation of mature chondrocytes and culture them *in vitro*, which inevitably has limitations such as limited cells, *in vitro* chondrocyte dedifferentiation, and donor-site morbidity caused by cartilage harvest(Zellner et al., 2017). The potential of stem cells to overcome the limitations of the current methods for cartilage regeneration has attracted widespread interest.

Human embryonic stem cells (hESCs) are considered to be a promising cell source for cartilage regeneration because of their unlimited self-renewal capacity and multidirectional differentiation potential (Toh et al., 2011). As the field of stem cells continues to expand, more stem cell types are being discovered. Currently, the majority of cartilage regeneration research focuses on MSCs, which can be obtained from mesenchymal tissues and have the capacity to proliferate and differentiate into chondrocytes (Li et al., 2018; Wang et al., 2019). Clinical trials are systematic studies conducted in humans and are indispensable for determining efficacy and safety. The effectiveness of clinical trials is a prerequisite for further clinical applications. Clinical trials focusing on the potential of stem cells in cartilage regeneration have also been carried out (Wells et al., 2020). Some types of stem cells have already entered clinical trials, among which the hUCB-MSC-based therapeutic product CARTISTEM has been approved by the Korean FDA for marketing. Regenerative strategies for AC are the focus of study, and the scientific community (as well as researchers, research institutions, etc.) is constantly exploring this field; therefore, it is urgent to summarize and analyse the trends of this field.

The literature is the carrier of scientific progress. Bibliometric analysis uses mathematical and statistical methods to quantitatively and quantitatively analyse publications in medical databases, revealing the development history, research focus and future trends of a certain field. In addition, bibliometric analysis can implement comparisons between different countries or institutions by analysing the quality and quantity of previous research (Baldiotti et al., 2020; Yan et al., 2020). As much progress has been made concerning the potential of stem cells in cartilage regeneration, the number of related papers has also increased drastically, but related bibliometric studies have not been performed. In this study, we analysed publications about the use of stem cells for cartilage regeneration in the Web of Science (WoS) database during 2010–2020. We conducted statistical analysis on the number of publications, keyword frequency, and citation frequency. We conducted qualitative and quantitative analyses of research from different countries, institutions, journals and authors. We also summarized previous clinical trials. We aim to map thematic trends and hotspots related to the capability of stem cells for cartilage regeneration, providing a reference for future research.

## 2 Material and Methods

### 2.1 Data Acquisition and Search Strategy

**FIGURE 1:**
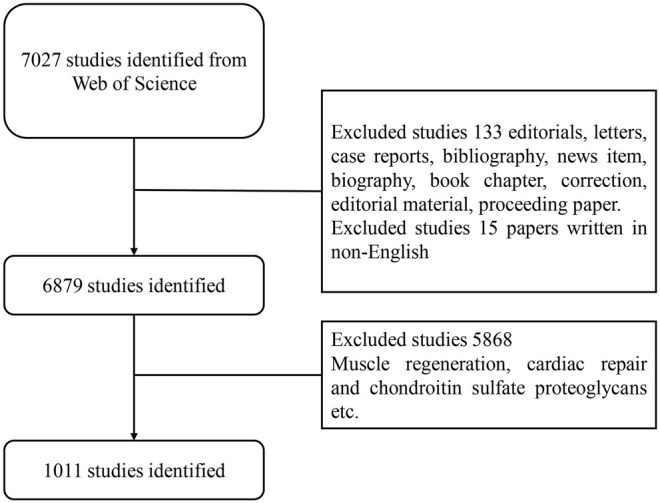
Flow chart of the screening process for research on stem cells for cartilage.

Web of Science is an important academic database worldwide that has been widely used in bibliometric analysis. We selected WoS as a data source for a comprehensive search on the utilization of stem cells for cartilage regeneration from 2010 to 2020. The search strategies were as follows: TS = (stem cell* OR SC) and (*cartilage or chondro*) and regenera* AND language: (English). The screening process is shown in Figure 1. Relevant clinical trial data were obtained from ClinicalTrials.gov (https://clinicaltrials.gov/), the keywords were the same, and the limited condition was “completed study”. All data were obtained online, and no ethical certification was required. All searches were conducted on November 27, 2020, to avoid bias related to database updates.

### 2.2 Data Collection

Two authors (Demeng Xia and Jianghong Wu) independently screened and extracted all data from the final included articles, including titles, keywords, authors, institutions, journals, dates of publication, countries/regions, citations, H-index, etc. We then conducted analysis with Microsoft Excel 2016, CiteSpace version V and VOSviewer.

### 2.3 Bibliometric Analysis

Relative research interest (RRI) is an indicator of activity in a certain field, and RRI is defined by weighted publications per year (WPPY) in a certain field and all weighted publications per year in PubMed (AWPPY). We used Excel to calculate the RRI and to display the time curve. The impact factor (IF) was acquired in the newest edition of the journal citation report (JCR). The visualization software programs VOSviewer and CiteSpace are widely used in bibliometric analysis. We employed VOSviewer software to perform coupling analysis of nations and institutions, co-citation analysis of journals, and co-occurrence of key words. The average appearing year (AAY) was used to describe the relative novelty of keywords. We employed CiteSpace mainly to perform co-citation analysis of references.

## 3 Result

### 3.1 Field Activity and Global Contributions

#### 3.1.1 Field Activity Analysis

**FIGURE 2:**
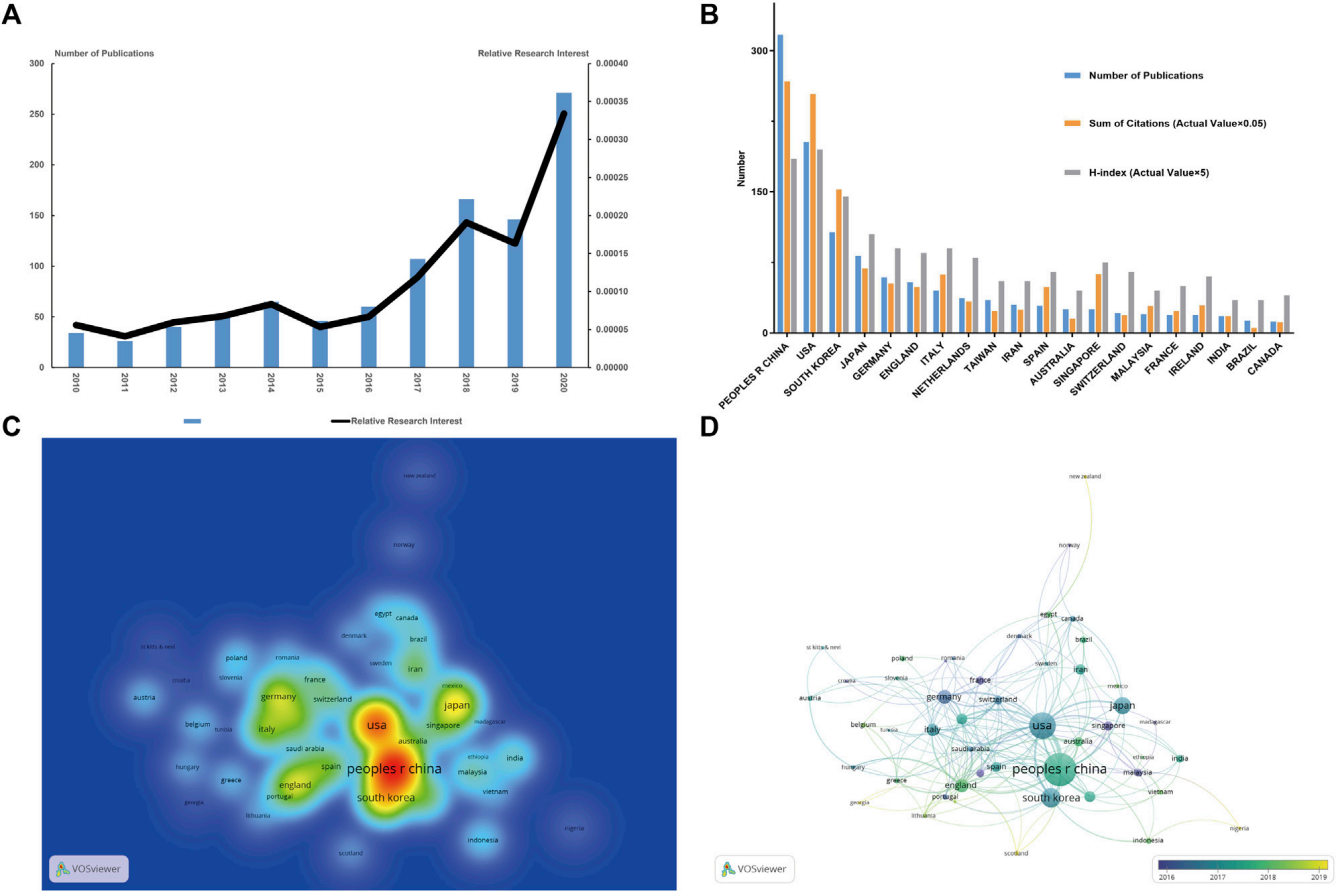
Articles related to stem cells for cartilage regeneration published worldwide**. (A)** Annual publications worldwide and the relative research interest (RRI) for stem cells for cartilage regeneration. **(B)** The number of publications, citation frequency (actual value × 0.05), and H-index (actual value × 5) in the top 20 countries or regions. **(C)** Density map of regional distribution of articles based on VOSviewer. **(D)** Collaboration between countries or regions based on VOSviewer.

Depending on the inclusion criteria, a total of 1,011 articles related to stem cells for cartilage regeneration were included in the final analysis. According to the annual distribution of publications, although there was a slight decline in 2010–2011 (35–26 publications per year), 2014–2015 (65–46 publications per year) and 2018–2019 (166–146 publications per year), the overall trend of the publication output increased from 2010 to 2020 (34–271 publications per year). The trend of the RRI was similar, suggesting that the field of stem cells for cartilage regeneration is receiving more attention in general (Figure 2A).

#### 3.1.2 Global Contribution to the Field

According to the national distribution of publications, China (317 publications) is the most productive country, followed by the United States (203 publications), South Korea (107 publications), Japan (82 publications) and Germany (59 publications) (Figure 2B). It is more intuitive to identify these publishing centres from the density map (Figure 2C). The top 20 countries with the most publications are listed in Table 1.

**TABLE 1:** The top 20 countries that contributed publications on stem cells for cartilage regeneration.

Country	No. of publications	Sum of citations	Citations	H-index
Peoples r China	317	5,351	4,993	37
USA	203	5,081	4,903	39
South Korea	107	3,048	2,938	29
Japan	82	1,374	1,313	21
Germany	59	1,054	1,041	18
England	54	974	949	17
Italy	45	1,244	1,223	18
Netherlands	37	668	652	16
Taiwan	35	467	457	11
Iran	30	493	478	11
Spain	29	974	960	13
Australia	25	304	294	9
Singapore	25	1,253	1,216	15
Switzerland	21	375	372	13
Malaysia	20	577	562	9
France	19	463	461	10
Ireland	19	585	575	12
Iindia	18	360	347	7
Brazil	13	109	107	7
Canada	12	230	229	8

In terms of total citations, the top four countries are China (5,351 citations), the United States (5,081 citations), South Korea (3,048 citations) and Japan (1,374 citations). Singapore is not among in the top 10 productive countries (25 publications) but ranks fifth for total citations (1,253 citations). The countries with the highest H-index are as follows: first, the United States (39); second, China (37); third, South Korea (29); fourth, Japan (21); and fifth, Italy (18) (Figure 2B).

The collaborations between countries are shown in Figure 2D. The size of the circles indicates the number of publications, and the width of the connecting line between two circles indicates the degree of collaboration. The United States has the strongest total link strength, which means that the United States has the predominant influence in this field. Many countries have concentrated years of article output. France, Germany and Singapore had the most publications concentrated before 2016. Then, the U.S. and Japan intensively published articles during the next 2 years. Articles from England and mainland China were mainly published after 2018.

#### 3.1.3 Analysis of Institution Distribution

The four institutions with the most publications are all located in China, and sequentially, they are Shanghai Jiao Tong University (34 publications), Peking University (27 publications), Sichuan University (26 publications), and Chinese PLA General Hospital (22 publications). The Chinese Academy of Science (21 publications) and National University of Singapore (21 publications) tied for fifth. The top 20 institutions with the most publications are listed in Table 2.

**TABLE 2:** Top 20 institutions with the most publications in the field of stem cells for cartilage regeneration.

Institution	Country	No. of publications	No. of citations
Shanghai jiao tong university	CHINA	34	4,120
Peking universty	CHINA	27	285
Sichuan university	CHINA	26	452
Chinese people’s liberation army general hospital	CHINA	22	445
Chinese academy of sciences	CHINA	21	218
National university of singapore	Singapore	21	1,051
Chinse university of hong kong	China	20	406
Zhejiang university	China	20	478
Sun yat sen university	China	18	490
Nanjing medical university	China	17	132
Pennsyllvania commonwealth system of higher education pcshe	USA	17	463
Tokya medical dental university tmdu	Japan	16	480
University of twente	Netherlands	16	399
Central south university	China	15	121
Kyoto university	Japan	15	257
Fudan university	China	14	181
Harvard university	USA	14	824
Institutnational de la sante et de la recherche medicale inserm	France	14	319
University of pittsburgh	USA	14	458
Wst virginia university	USA	14	315

**FIGURE 3:**
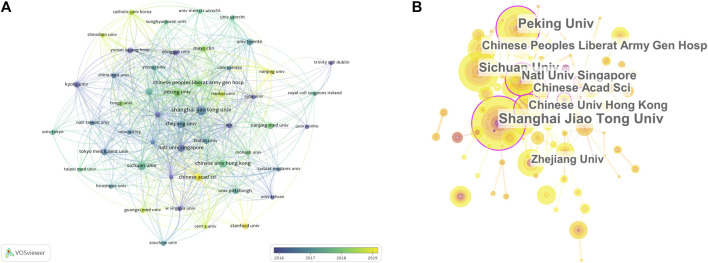
Articles on stem cells for cartilage regeneration published at different institutions**. (A)** Collaboration between institutions based on VOSviewer **(B)** cooperation network analysis of institutions based on CiteSpace.

Figure 3A highlights the close and complex collaborative relations between different institutions. CiteSpace was employed to analyse the centrality of institutions. The purple circle indicates centrality, and the area of the circle is proportional to the centrality. Shanghai Jiao Tong University and National University of Singapore are the most prominent institutions, which suggests that they are regarded as pivotal points (Figure 3B).

#### 3.1.3 Analysis of Journal Distribution

**FIGURE 4:**
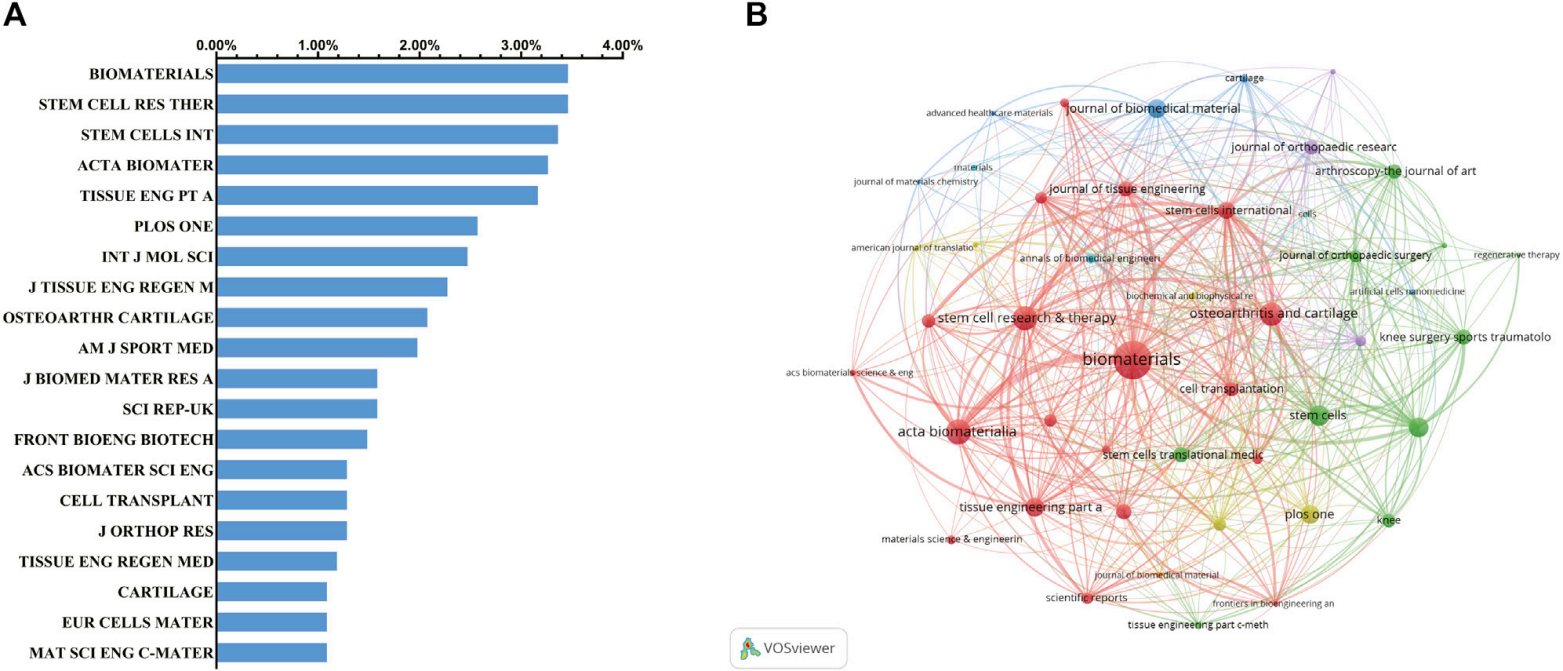
Articles published in different journals on stem cells for cartilage regeneration. **(A)** Top 20 journals that produced the largest number of articles. **(B)** Cocitation analysis of journals based on VOSviewer.

Different journals include different fields of publication. Therefore, we performed a journal distribution analysis of publications on the use of stem cells for cartilage regeneration. The journals *Biomaterials* (impact factor = 10.317, 2019) and *Stem Cell Research and Therapy* (impact factor = 5.116, 2019) published the most studies, with 35 publications each. There were 34 articles on stem cell research for cartilage regeneration in *Stem Cells International* (IF = 3.869, 2019), 33 articles in *Acta Biomaterialia* (IF = 7.242, 2019) and 32 articles in *Journal of Tissue Engineering Part A* (IF = 3.776, 2019). The top 20 journals with the most publications are listed in Figure 4A.

The journals are not kept in isolation. *Biomaterials* and *Acta Biomaterialia* have the closest relationship. As far as this field is concerned, journals with a high volume of publications also have high centrality (Figure 4B).

### 3.2 Overview of Landmark Articles and Authors

The authors with the highest number of publications from 2010 to 2020 are shown in Table 3, and the total citations of the publications are also listed. SEKIYA I and YANG F tied for first place. However, TOH WS of the National University of Singapore is particularly noteworthy. Although TOH WS published only 10 articles, the total number of citations reached 740, contributing greatly to Singapore’s total citations. This shows that his viewpoint has drawn widespread attention in the field.

**TABLE 3:** Top 10 authors in the field of stem cells for cartilage regeneration ranked by publication number.

Author	Country	Affiliation	No. of publications	No. of citations
Sekiya i I	Japan	Tokyo medical and dental university	14	387
Yang F	USA	Stanford university	14	52
Guo oy	Peoples r china	Chinese people’s liberation army general hospital	13	229
Pei m	USA	West virginia university	13	276
Peng j	Peoples r china	Chinese people’s liberation army general hospital	12	191
Zhang x	Peoples r china	Peking university	11	146
Koh yg	South korea	Yonsei sarang hosp	10	240
Toh ws	Singpore	National university of singapore	10	740
Mobasheri a	Finland	University of oulu	10	502
Tuan rs	Peoples r china	Chinese university of hong kong	10	342

**FIGURE 5:**
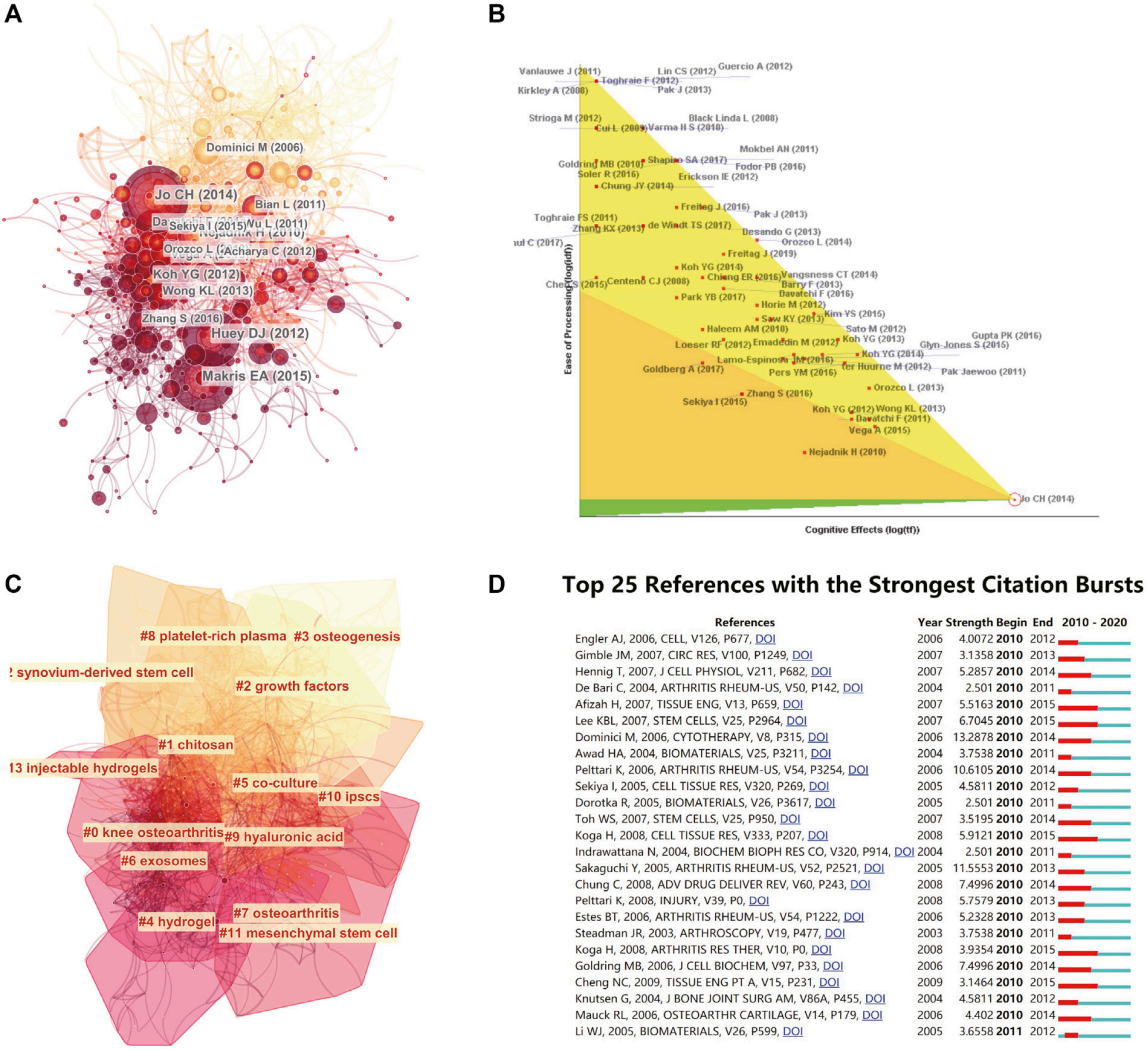
Mapping of references in studies on stem cells for cartilage regeneration. **(A)** A simplified co-citation network of references about stem cells for cartilage regeneration based on CiteSpace (burst references are represented by red nodes). **(B)** The article with the highest citation rate was also processed for in-depth analysis. **(C)** Clustering analysis of the co-citation network based on CiteSpace. **(D)** Top 25 references with strongest citation bursts based on CiteSpace.

We analysed the references of the shortlisted publications and constructed the cocitation network. Jo Ch’s article (doi:10.1002/stem.1634) published in *Stem Cells* in 2014 is at a key node in the co-citation network (Figure 5A). We further performed an in-depth analysis of the article with the highest citation rate (Figure 5B). Then, we implemented a cluster analysis. The references in the co-citation network are divided into 12 diverse clusters (Figure 5C). The clusters are labelled by extricating terms from the titles of the cited publications. From a timeline perspective, the theoretical basis of earlier publications is mainly osteogenesis and growth factors. Articles in recent years have been based on hydrogels and mesenchymal stem cells. The top 10 articles with the most total citations are detailed in Table 4.

**TABLE 4:** Top 10 references with the most citations in the field of stem cells for cartilage regeneration.

Title	Corresponding author	Journal	If	Publication year	Total citations
Intra-articular injection of mesenchymal stem cells for the treatment of osteoarthritis of the knee: A proof-of-concept clinical trial	Yoon, KS	STEM CELLS	6.523	2014	424
Isolation of adipose-derived stem cells and their induction to a chondrogenic phenotype	Guilak, F	NATURE PROTOCOLS	8.326	2010	291
Chitosan, hyaluronan and chondroitin sulfate in tissue engineering for cartilage regeneration: A review	Muzzarelli, RAA	CARBOHYDRATE POLYMERS	3.479	2012	267
Treatment of knee osteoarthritis with allogeneic bone marrow mesenchymal stem cells: A randomized controlled trial	Garcia-Sancho, J	TRANSPLANTATION	3.69	2015	258
Treatment of knee osteoarthritis with autologous Mesenchymal Stem Cells: A Pilot Study	Garcia-Sancho, J	TRANSPLANTATION	3.535	2013	251
Infrapatellar fat pad-derived mesenchymal stem cell therapy for knee osteoarthritis	Choi, YJ	KNEE	3.781	2012	232
Exosomes derived from human embryonic mesenchymal stem cells promote osteochondral regeneration	Toh, WS	OSTEOARTHRITIS AND CARTILAGE	4.742	2016	230
Hydrogels that mimic developmentally relevant matrix and N-cadherin interactions enhance MSC chondrogenesis	Burdick, JA	PROCEEDINGS OF THE NATIONAL ACADEMY OF SCIENCES OF THE UNITED STATES OF AMERICA	9.809	2013	223
Exosomes derived from miR-140-5p-overexpressing human synovial mesenchymal stem cells enhance cartilage tissue regeneration and prevent osteoarthritis of the knee in a rat model	Zhang, CQ	THERANOSTICS	8.063	2017	197
Comparative evaluation of MSCs from bone marrow and adipose tissue seeded in PRP-derived scaffold for cartilage regeneration	Zhang, CQ	BIOMATERIALS	7.604	2012	179

The burst detection function in CiteSpace is helpful to understanding the research frontiers. The top 25 strongest citation bursts of the references are presented, and there was no article with a sudden change in the number of citations in the past 3 years (Figure 5D).

### 3.3 Co-Occurrence Analysis of Key Words

**FIGURE 6:**
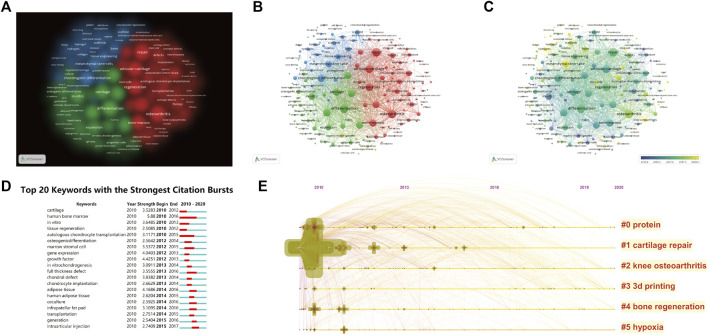
Mapping of keywords in studies on stem cells for cartilage regeneration. **(A)** Clustering analysis of key words based on VOSviewer. **(B)** Network visualization of keywords based on VOSviewer. **(C)** Chronological order of keywords based on VOSviewer. **(D)** Top 20 keywords with the strongest citation bursts based on CiteSpace. **(E)** Keyword timeline visualization from 2010 to 2020.

Keywords represent the main topic of publications. Co-occurrence analysis of keywords is conducive to systematically understanding the relationship between keywords and consequently grasping the relationship between various topics in this field. Further cluster analysis helps to systematically understand the current progress in this field. VOSviewer was employed to analyse keywords (defined as words that were used more than five times in titles and abstracts in all publications) in all included publications. A total of 203 identified keywords were mainly divided into three clusters: ‘clinical study,’ ‘tissue engineering’ and ‘biological study’. In clinical studies, the keywords with the highest frequency were repair (273 times), articular cartilage (238 times), osteoarthritis (264 times), regeneration (222 times) and bone marrow (193 times). In tissue engineering, these words were chondrogenic differentiation (203 times), scaffold* (160 times), mesenchymal stem cells (152 times), tissue engineering (108 times) and bone (97 times). In biological studies, the words were differentiation (282 times), chondrogenesis (208 times), cartilage (196 times), tissue (191 times) and chondrocytes (186 times) (Figures 6A,B). The annual distribution of keywords is indicated. Chronological order is presented from dark blue to bright yellow. In “Clinical studies,” the earliest keyword was “autologous chondrocyte transplantation” (AAY 2014.7143), with 14 instances, and the latest word was “double-blind” (AAY 2018.75), with 8 times. In tissue engineering, the earliest word was “growth factors” (AAY 2014.125), with 8 times, and the latest word was “3D printing” (AAY 2019.625), with nine instances. In the biological study, the earliest word was “cartilage formation” (AAY 2014.875), with 8 times, and the latest word was “extracellular vesicles” (AAY 2018.9091), with 12 times (Figure 6C). According to the colour intensity, we found that the research hotspot on stem cells for cartilage regeneration gradually shifted to tissue engineering in recent years (Table 5, Figure 5). The 20 keywords with the strongest citation burst are listed in Figure 6D, and no keywords exhibited any sudden changes in the number of citations over the past 3 years.

**TABLE 5:** Summary of clinical trials on stem cells for cartilage regeneration.

Study	ClinicalTrials.gov Identifier	Office title	Time	Country	Design	No. of patients	Conditions	Intervention		Phase	Primary outcome measurement	Primary purpose	Summary
								Treatment group	Comparison group				
1	NCT02118519	Use of Mesenchymal Stem Cells for the Repair of Articular Cartilage Disorder of Knee Using Intra-articular Injection of Mesenchymal Cells Alone or Mesenchymal Cells With Platelet Lysate	2014.01-2015.08	Jordan	Non-Randomized, Parallel Assignment, Double (Participant, Investigator)	13	Articular Cartilage Disorder of Knee; Osteoarthritis	Biological: Autologous mesenchymal stem cells	No	Phase 2	Assessing the therapeutic benefits of the injected Autologous Mesenchymal Stem Cells	Treatment	This study is to evaluate the induction of autologous repair chondrogenesis to regenerate injured articular cartilage using bone marrow mesenchymal stem cells (MSCs) after in vitro expansion under restricted culturing conditions.
2	NCT02291926	Phase I Study of Human Umbilical Cord Mesenchymal Stem Cell Implantation in the Treatment of Articular Cartilage Defect of Knee	2014.12-2016.12	China	Single Group Assignment, None (Open Label)	20	Cartilage Diseases; Osteoarthritis	Biological: Human umbilical cord mesenchymal stem cells	No	Phase 1	Severity of adverse events	Treatment	The purpose of this study is to evaluate the safety and efficacy of human umbilical cord mesenchymal stem cell(hUC-MSC) for articular cartilage defect of knee.
3	NCT01076673	Articular Cartilage Regeneration With Autologous Peripheral Blood Stem Cells Versus Hyaluronic Acid: A Randomized, Controlled Trial	2009.10-2012.05	Malaysia	Randomized, Parallel Assignment, None (Open Label)	50	Articular Cartilage Disorder of Knee	Biological: Peripheral blood stem cells	Drug: Hyaluronic acid	Phase 2	Change in tissue histology after subchondral drilling surgery using serial MRI scanning and cartilage biopsies	Treatment	The purpose of this study was to compare histologic and MRI evaluation of articular cartilage regeneration in patients with chondral lesions treated by arthroscopic subchondral drilling followed by postoperative intra-articular injections of hyaluronic acid (HA) with and without peripheral blood stem cells (PBSC).
4	NCT00850187	Treatment of Full-thickness Articular Cartilage Defects in the Knee of Patients With Autologous Bone-marrow Mesenchymal Stem Cells and Scaffold	2008.08-2010.12	Iran	Single Group Assignment, None (Open Label)	6	Knee Osteoarthritis	Biological: Bone marrow derived mesenchymal stem cells	No	Phase 1	Knee cartilage defects	Treatment	The purpose of this study is to investigate the efficacy and safety of autologous transplantation of Bone Marrow Mesenchymal stem cells (MSCs) mixed with collagen I scaffold in patient with Knee cartilage defects and osteoarthritis.
5	NCT01895413	Autologous Bone Marrow-derived Mesenchymal Stem Cells Used in the Treatment of Articular Cartilage Injury	2012.09-2016.01	Brazil	Single Group Assignment, None (Open Label)	10	Osteoarthritis	Procedure: Bone marrow aspiration	No	Phase1, Phase 2	Change in WOMAC (Western Ontario and McMaster Universities) score	Treatment	This is a non-randomized study aimed to determine the safety and efficacy of intra-articular injection of autologous bone marrow-derived mesenchymal stem cells in patients with knee articular cartilage defects.
6	NCT01227694	Articular Cartilage Regeneration in Gonarthrosis Grade II and III by Articular Infiltration of Xcel-m-condro-alpha.	2010.10-2013.01	Spain	Single Group Assignment, None (Open Label)	15	Knee Osteoarthritis; Knee Injuries; Joint Diseases; Rheumatic Diseases; Cartilage Diseases	Other: Autologous MSC (mesenchymal stem cells) knee implantation	No	Phase1, Phase 3	Feasibility of autologous bone marrow mesenchymal stem cells (MSC) knee articular infiltration	Treatment	This is a prospective, open-label, single-dose, single-arm phase I-II study in which 15 patients diagnosed with gonarthrosis grade II-III (Kellgren and Lawrence) will enter the study with the primary objective of assessing the feasibility and safety of the knee articular infiltration of autologous bone marrow mesenchymal stem cells (MSC). Secondary objectives are to assess the efficacy by imaging procedures and clinical questionnaires.
7	NCT01733186	Evaluation of Safety and Exploratory Efficacy of CARTISTEM®, a Cell Therapy Product for Articular Cartilage Defects: A Phase I/IIa Clinical Trial in Patients With Focal, Full-thickness Grade 3-4 Articular Cartilage Defects of the Knee	2013.01-2017.08	United States	Single Group Assignment, None (Open Label)	12	Degeneration Articular Cartilage Knee	Biological: CARTISTEM (a cell therapeutic product)	No	Phase1, Phase 4	Number of adverse events	Treatment	The purpose of this study is to determine whether CARTISTEM, a cell therapeutic product, is safe and effective in the treatment of articular cartilage defects of the knee as a result of ageing, trauma, or degenerative diseases.
8	NCT01207661	Mesenchymal Stem Cells Transplantation for Articular Cartilage Resurfacing in Patient With Osteoarthritis of Knee Joint	2009.08-2010.11	Iran	Single Group Assignment, None (Open Label)	6	Osteoarthritis	Biological: Mesenchymal injection	No	Phase 1	Pain relief	Treatment	This study is designed to evaluate therapeutic potential and safety of mesenchymal stem cells in improvement of osteoarthritis clinical manifestations.This study is designed to evaluate therapeutic potential and safety of mesenchymal stem cells in improvement of osteoarthritis clinical manifestations.
9	NCT01879046	Regenerative Medicine of Articular Cartilage: Characterization and Comparison of Chondrogenic Potential and Immunomodulatory Adult Mesenchymal Stem Cells	2015.10-2016.12	France	Single Group Assignment, None (Open Label)	35	Knee Osteoarthritis	Procedure: Blood, bone marrow, synovial fluid and Hoffa's fat pad samplings achieved by arthroplasty	No	Not Applicable	Increased expression of chondrogenic markers	Other	This project aims to determine the most appropriate source for regenerative medicine of cartilage stem cells from tissue taken during arthroplasty in patients with osteoarthritis.
10	NCT01041001	Randomized, Open-Label, Multi-Center and Phase 3 Clinical Trial to Compare the Efficacy and Safety of Cartistem® and Microfracture in Patients With Knee Articular Cartilage Injury or Defect	2009.02-2011.01	Republic of Korea	Randomized, Parallel Assignment, None (Open Label)	104	Cartilage InjuryOsteoarthritis	Biological: CARTISTEM (a cell therapeutic product)	Procedure: Microfracture treatment	Phase 3	ICRS Cartilage Repair Assessment will follow to determine the appropriate grade. The treatment will be considered efficacious if the ICRS grade drops by at least 1 grade or more from baseline to week 48.	Treatment	The purpose of the study is to assess and compare the safety and efficacy of the allogeneic-unrelated umbilical cord blood-derived mesenchymal stem cell product (Cartistem®) to that of a microfracture treatment in patients with articular cartilage defect or injury.
11	NCT01873625	Resurfacing Articular Cartilage With Mesenchymal Stem Cells Transplantation in Patients With Knee Joint Osteoarthritis Affected by Rheumatoid Arthritis: Randomized Triple Blind Clinical Trial Phase II/III (ACRCT)	2009.10-2011.12	Iran	Randomized, Parallel Assignment, None (Open Label)	60	Rheumatoid Arthritis	Biological: mesenchymal cell transplantation	Biological: placebo	Phase 2, Phase 3	Pain relief	Treatment	Patients are treated initially by pain management. In patients who don't response to first line treatment invasive treatment like total knee replacement is done. The investigators designed this clinical study with the aim of evaluating therapeutic effects of intra-articular injection of bone marrow mesenchymal stem cells (BM-MSCs) in 60 patients with knee osteoarthritis.
12	NCT01183728	Regeneration of Articular Cartilage in Grade II, III and IV Knee Osteoarthritis by Intraarticular Injection of Autologous Bone Marrow Stem Cells Expanded ex Vivo With a GMP Procedure Developed by IBGM-Valladolid (MSV)	2010.05-2014.09	Spain	Single Group Assignment, None (Open Label)	12	Knee Osteoarthritis; Knee Degenerative Disease	Biological: Autologous bone marrow mesenchymal stem cells (MSV)	No	Phase 1, Phase 2	Feasibility and Safety of the Implementation of MSV in the Treatment of Osteoarthritis of the Knee.	Treatment	The working hypothesis proposes that MSV antiinflammatory effect will help healing of articular cartilage degeneration to a grade enough to be objectivized by questionnaires and imaging procedures. In this prospective study, the researchers aim to evaluate the feasibility and safety of the implantation of 40 millions MSV in knees with osteoarthritis of grade II-IV (Kellgren and Lawrence).
13	NCT01626677	Long Term Follow-Up Study of CARTISTEM® Versus Microfracture for the Treatment of Knee	2012.06-2015.05	Republic of Korea	Randomized, Parallel Assignment, None (Open Label)	103	Degenerative OsteoarthritisDefect of Articular Cartilage	Biological: CARTISTEM (a cell therapeutic product)	Procedure: Microfracture	Phase 3	Degree of improvement in knee assessments compared to the active control (microfracture)	Treatment	This is a long term follow-up study to investigate the safety and efficacy of CARTISTEM®, human umbilical cord blood-derived mesenchymal stem cells, in repair of cartilage injury or defects, compared with microfracture. Subjects who participated in and completed the Phase III trial (NCT01041001) will be tracked until the 60 month post-treatment timepoint.
14	NCT02142842	Phase I/II Study of Transplantation of Autologous Adipose Stem Cells and Activated Platelet Rich Plasma in Knee Osteoarthritis Treatment	2013.04-2015.12	Vietnam	Randomized, Parallel Assignment, Single (Investigator)	30	Knee Osteoarthritis	Biological: Autologous adipose tissue stromal vascular fraction	Biological: platelet rich plasma	Phase 1, Phase 2	Number of participants with adverse events	Treatment	To evaluate the function of regenerating the injured cartilage by adipose stem cells and platelet rich plasma.
15	NCT02219113	Effectiveness and Safety of Intraarticular Administration of Autologous Adipose-Derived Regenerative Cells for Treatment of Degenerative Damage of Knee Articular Cartilage	2014.07-2017.04	Russian Federation	Single Group Assignment, None (Open Label)	12	Knee Joint Osteoarthritis	Procedure: Arthroscopic surgery; Procedure: Liposuction; Device: ADRC isolation; Other: Intra articular administration of autologous ADRC	No	Phase 1, Phase 2	Number of serious adverse events (SAEs) and serious adverse reactions (SARs)	Treatment	Autologous adipose-derived regenerative cells (ADRC) extracted using Celution 800/CRS System (Cytori Therapeutics Inc) from a portion of the fat harvested from the patient's front abdominal wall. ADRC will be administered one-time intraarticularly. This is a single arm study with no control. All patients receive cell therapy.
16	NCT02037204	Instant MSC Product Accompanying Autologous Chondron Transplantation (IMPACT): Safety and Feasibility of a Single-stage Procedure for Focal Cartilage Lesions of the Knee.	2013.03-2016.04	Netherlands	Single Group Assignment, None (Open Label)	35	Foreign-Body Reaction; Inflammation; Effusion (L) Knee; Knee Pain Swelling	Other: Cartilage repair surgery	No	Phase 1, Phase 2	Safety: Adverse Events	Treatment	This is a phase I/II prospective monocenter study to evaluate the safety and feasibility of the IMPACT for treatment of focal articular cartilage lesions of the knee.

### 3.4 Clinical Research Summary

*ClinicalTrials.gov (* *https://clinicaltrials.gov/* *)* is one of the most important databases of clinical studies conducted around the world, and it was developed by the National Library of Medicine (NML) and the U.S. Food and Drug Administration (FDA). *ClinicalTrials* could provide clinical trial information inquiry services of clinical trial information to the general public and could also provide clinical trial registration services to medical researchers and institutions. We used *ClinicalTrials.gov* to list the currently completed clinical studies on stem cells for cartilage regeneration (Table 5). A total of 16 clinical trials have been completed, and among them, CARTISTEM has been approved by the Korean FDA. The 16 clinical trials came from 12 countries, with the top three countries being Iran (3), Republic of Korea 2) and Spain (2). Less than one-third of clinical trials have a comparison group, and more than two-thirds have not yet entered Phase 3. The overall size of the included samples is relatively small. Only NCT01041001 and NCT01626677 exceed 100, both of which are from the Republic of Korea and relevant to CARTISTEM. NCT01041001 assesses and compares the safety and efficacy of the allogeneic-unrelated umbilical cord blood-derived mesenchymal stem cell product (CARTISTEM) to that of a microfracture treatment, and NCT01626677 is a long-term follow-up study to investigate its safety and efficacy. In addition, the most important condition of these clinical trials is knee-related cartilage regeneration, which may be related to its high incidence and feasibility for performing clinical research. From the perspective of article publication, only 6 projects have obtained results.

## 4 Discussion

This study offered a bibliometric analysis of stem cells for cartilage regeneration from 2010 to 2020. With the development of bibliometric software, bibliometric analysis is now widely pursued. It can help beginners to understand the development process and trends of a specific field intuitively and systematically. It is also beneficial to find milestone achievements and new research hotspots.

Regenerative therapy uses multidisciplinary knowledge and technology to achieve regeneration of damaged tissues or organs. Regenerative therapy integrates knowledge and technology from multiple disciplines, aiming to regenerate damaged tissues or organs (Zhang et al., 2016; El-Jawhari et al., 2017). The number of publications and RRI in stem cells for cartilage regeneration have been on the rise, especially in the past 5 years, suggesting that the proportion of this field among all academic fields is increasing (Figure 2A). In addition, more than half of the top five journals in this field have an impact factor of five or more (Figure 4), which means that the topic of stem cells for cartilage regeneration has drawn widespread attention according to the method used to calculate the impact factor. Therefore, more in-depth studies will be published in the future and prove the feasibility and application prospects of stem cells for cartilage.

In terms of national and regional distribution, the total number of publications and citations of China ranks first in the world. However, the United States still ranked first in terms of the H-index, indicating its predominant influence (Figures 2B,D). When we further analyse the average citation rate (only comparing countries with more than 100 publications), the average citation rate of China is 16.88, while those of the United States and South Korea are 25.03 and 28.49, respectively (Table 1). The influence and contribution of scientific institutions represent a fundamentally important aspect of a country or region. In terms of total publications, 9 of the top 10 institutions are located in China, accounting for 70% of the total publication number. However, among the top 5 institutions in China in terms of publication volume, none of the top 5 in the world in terms of total citations (Table 2; Figure 3). Although the citation rate of the articles are not completely equal to the importance of academic achievements, practical problems may be indicated when low citation rates becomes a common phenomena. From the perspective of time distribution, the concentrated years of article output of China were approximately 2018 (Figure 2D). However, there were no keywords or references with any sudden change in the number of citations in the past 3 years (Figures 5D, 6D). This discrepancy suggests that scientific achievements in China have not formed a hot topic in this field. These results indicate that most academic achievements are the completion and supplementation of previous breakthrough results instead of original creative discoveries and techniques. The National Natural Science Foundation of China (NSFC) is one of the main channels for supporting basic research in China, and a series of changes in the NSFC application rules have taken place in recent years, aiming to improve the quality and innovativeness of publications. We hypothesize that the centrality of China will gradually increase in the future.

As far as authors are concerned, SEKIYA I (Tokyo Medical and Dental University, JAPAN) and YANG F (Stanford University, United States) are the most productive. However, in terms of the number of citations, the data of Toh WS (National University of Singapore, Singapore) are particularly attractive (Table 3). He has published 10 papers on stem cells for cartilage regeneration, but the total number of citations has reached 740. The total citations of Toh WS accounted for approximately 60% of Singapore and 70% of National University of Singapore in the study (Table 1, Table 2). In 2007, Toh WS successfully constructed an experimental system in which human embryoid body (EB)-derived cells could directly differentiate into chondrocytes under specific culture conditions (Toh et al., 2007). In 2009, Toh WS and his team improved this high-density micromass model system. Through using a more effective combination of growth factors and extracellular matrix substrate, they isolated a highly expandable and homogenous chondrogenic cell population named TC1 (Toh et al., 2009). In 2010, Toh WS and his team constructed hESC-derived chondrogenic cell-engineered cartilage (HCCEC) from the abovementioned cell population in the culture of hyaluronic acid (HA)-based hydrogel and verified its long-term viability and safety in a rat model, providing a practical strategy of applying hESCs for cartilage regeneration (Toh et al., 2010). Afterwards, Toh WS and his team shifted the focus of research to mesenchymal stem cell-derived exosomes and illustrated the underlying mechanisms of MSC exosomes in the biological behaviour of chondrocytes, extracellular matrix homeostasis, and immune reactivity in cartilage regeneration (Toh et al., 2017; Zhang et al., 2018). The achievements in MSC-derived exosomes study grapes attention of scientists around the world, and the references in this place had more than 100 citations. In addition, the author and his team published three reviews and summarized the potential and perspective of human embryonic stem cells (ESCs) in cartilage tissue engineering and regenerative medicine, the interactions between stem cells and extracellular matrix for cartilage regeneration, and the potential and perspective of MSC exosomes in cartilage regeneration.

As a key node in the co-citation network, Jo Ch’s article (doi:10.1002/stem.1634) not only had the largest number of citations (Table 4) but also had the highest impact (Figures 5A,B). Jo Ch is the first author, and the corresponding author of this article is Kang Sup Yoon. Kang Sup Yoon and his team substantiated the safety and efficacy of intra-articular injection of autologous AD-MSCs through clinical index, radiological evaluation, arthroscopic evaluation and histological evaluations. More importantly, Kang Sup Yoon and his team evaluated the effects of different doses of AD-MSCs injected into articular tissue. It is currently known that a sufficient number of MSCs can inhibit articular cartilage degradation and promote its regeneration (Ichiseki et al., 2018). However, when evaluating efficacy and safety comprehensively, the optimal cell dose needs to be clarified. Preliminarily exploring the therapeutic dose, Kang Sup Yoon and his team suggested that at least 1.0 × 10^8^ MSCs per injection would be an initial prerequisite for consistently good results (Jo et al., 2014). In conclusion, this proof-of-concept clinical trial confirmed the effectiveness of intra-articular injection of MSCs and presented a fair opinion on the injection dose, promising to encourage continued clinical research and application.

In the “Clinical studies,” the latest word was “double-blind” (AAY 2018.75), with 8 times (Figure 6C), which heralds future trends in the biological study of stem cells for cartilage regeneration. A double-blind trial is a test where neither the tester nor the testees know the exact group of testees, which helps to avoid cognitive bias (Cho et al., 2019). Due to its higher cost, there are very few double-blind trials in this field (Table 5). A double-blind trial from South Korea explored the effectiveness and safety of genetically engineered autologous chondrocytes, which were named TissueGene-C (TG-C), for knee osteoarthritis. TG-C was administered by a single intra-articular injection, and then subjective and objective assessments were performed according to a clinical rating scale and radiographic evaluation (Kim et al., 2018). Although subjective evaluation attests to the effectiveness of this treatment, changes in joint space widths, bone area and cartilage thicknesses only show a trend that are not statistically significant. This research has laid the foundation for the development of double-blind trials in this field. On the basis of reality and possibility, double-blind experiments in stem cells for cartilage regeneration should also be attempted.

In tissue engineering, the latest word was “3D printing” (AAY 2019.625), with 9 times (Figure 6C). 3D printing was proposed by Chuck Hull in 1983 and includes two main processes: computer-aided design (CAD) and 3D printing to form products. In the 2000s, 3D printing was used in the production of surgical models and later developed to be used in the production of live cell structures, including articular cartilage. There are also different strategies regarding the product formation process, inkjet printing, laser-assisted printing, and bioextrusion (Wu et al., 2018; Messaoudi et al., 2020). The materials used for 3D printing (bioink) are a hot and difficult point. Bioink mainly consists of two parts: biomaterials as scaffolds **(**second highest frequency keywords in tissue engineering clusters**)** and embedded cells (Kim M. K. et al., 2020). Biomaterials used for tissue engineering need to have biocompatibility, biodegradability, and porosity, and they are mainly divided into natural polymers (alginate, gelatine, chitosan, and hyaluronan) or synthetic polymers (PCL, PGA, PEG) at present (Shen et al., 2019). After being modified, natural polymers can be made into a more stable form, hydrogels, which have been widely used in 3D printing (Diloksumpan et al., 2020). Synthetic polymers could be combined or coated with hydrogels to enhance their biocompatibility. Biomaterials can play a variety of roles depending on the embedded cell types, including chondrogenic differentiation (highest frequency keywords in tissue engineering clusters).

As mentioned earlier, chondrocytes have limited expansion efficiency and potential for dedifferentiation *in vivo*. In contrast, the application of stem cells in tissue engineering is more promising. According to current research, the mechanism underlying cartilage regeneration in stem cells is mainly attributed to direct differentiation of chondrocytes and paracrine secretion (Iturriaga et al., 2018; Kim Y. et al., 2020). BM-MSCs are among the most commonly used cell types in tissue engineering (Table 5), and they can be induced into multiple cell types, including chondrocytes. AD-MSCs are easily accessible via minimally invasive procedures, and they have the characteristics of synthesizing more collagen than other MSC types. SF-MSCs and Dental pulp MSCs are also have unique advantages. (Tan and Hung, 2017; Kabir et al., 2020). In addition to biological materials and stem cell types, environmental conditions and differentiation-promoting factors are also key points for 3D printing technology. For example, the surrounding medium’s oxygen content affects the chondrogenic differentiation of MSCs(Portron et al., 2015; Anderson et al., 2016), and TGF-β1 can promote and stabilize the chondrogenic phenotype (Futrega et al., 2021). Overall, 3D printing is a microcosm of tissue engineering: whether it can be promoted clinically depends on many factors, but 3D printing clearly constitutes an important direction of future research.

In the biological study, the latest term was “extracellular vesicles” (AAY 2018.9091), with 12 times (Figure 6C). Extracellular vesicles are released from the cell surface to body fluid, and they can transfer multiple types of cargo, such as lipids, nucleic acids and proteins, thus contributing to intercellular communication (Allan et al., 2020). Exosomes, microvesicles (MVs) and apoptotic bodies are currently the most attractive categories of extracellular vesicles, and they are different in terms of generation mechanism and size (Lawson et al., 2016). According to publication numbers, exosomes have attracted more attention among researchers. Currently, exosomes have been identified from multiple MSCs, including those derived from AD-MSCs, BM-MSCs, US-MSCs, hESCs, and iPSCs (Zhang et al., 2018; Xu et al., 2020). When MSCs sense changes in the surrounding microenvironment, they can secrete exosomes to interact with other cells. Exosomes have been proven to regulate the biological behaviour of chondrocytes, to modulate immune responses and to restore homeostasis to the ECM, thereby promoting cartilage regeneration (Zhang et al., 2018; Tan et al., 2020). Although the therapeutic efficacy, biosafety, kinetics and biodistribution of MSC exosomes need to be studied in depth, the regenerative potency of MSC exosomes provides new perspectives for the development of a cell-free MSC therapy strategy for cartilage regeneration.

The purpose of clinical trials is to repair damaged articular cartilage. The application of stem cells is expected to replace traditional medical treatment and achieve the goal of radical cures without undergoing such a major operation as joint replacement. According to current clinical trials, except for CARTISTEM, which uses commercial hUCB-MSCs(Park et al., 2017), all other clinical trials use autologous stem cells. The entire process includes collection, *in vitro* expansion, and intra-articular injection (Al-Najar et al., 2017). Subjective clinical scores, imaging evidence, and histological evaluation are currently the most important evaluation methods. The effectiveness, feasibility and safety of the concept of using mesenchymal stem cells in cartilage repair have been verified (Table 5). However, there are still some issues that need to be resolved. First, the most suitable cell source necessitates further exploration. Except for CARTISTEM, all other clinical trials use autologous stem cells, and the most commonly used type is BM-MSCs. The horizontal comparison of different types of cells is still missing. Second, it is unclear whether MSCs mainly differentiate into chondrocytes directly or more through paracrine forms to promote cartilage formation. Third, the optimal therapeutic course and dose need to be further explored. NCT01207661 indicated that the effect of a single injection was improved in the first 12 months and decreased from the 12th month onward (Emadedin et al., 2015). NCT01873625 also explored the number of cells per injection and provided a reference value of 40 million (Shadmanfar et al., 2018). However, several concerns about the use of stem cells remain unsolved, particularly regarding side effects (Rivera-Izquierdo et al., 2019). For example, challenges of stem cell transplantation include poor survivability, migration of the transplanted cells, and adverse immune reactions (Dunn et al., 2019). What’s worse, the tumorigenic properties of stem cells have been reported, they not only possess intrinsic tumorigenic properties, but can be induced to possess tumorigenic properties (Patel et al., 2008). The last but not the least, ethical considerations for stem cell therapy cannot be ignored. In thus, the best course of treatment and therapeutic dose need more exploration. In addition, current clinical trials are mainly short-term follow-ups, typically only 24 months or less. Long-term efficacy and safety evaluations need further verification.

This study investigated publications on stem cells for cartilage regeneration extracted from the Web of Science database and discussed hot issues in this comprehensive and objective field. However, limitations are inevitable. First, we only enrolled publications in the language of English, which may cause some important non-English studies in this field to be overlooked. In addition, although keywords can reflect the subject of the article to a certain extent, the preferences of different authors when using keywords and the shortness of keywords will lead to bias in trend analysis.

## Conclusion

Overall, this study summarized and analysed the global research trends concerning the use of stem cells for cartilage regeneration. The annual output of related publications has grown remarkably, and stem cells have positive development prospects for cartilage regeneration. China and the United States have made the largest contributions in this field. 3D printing, extracellular vesicles, and double-blind clinical trials are research hotspots and which are likely to be promising in the near future. Clinical trials with larger sample sizes and longer follow-up periods are needed for clinical transformation.

## Data Availability

The original contributions presented in the study are included in the article/Supplementary Material, further inquiries can be directed to the corresponding authors.
