# Supplementary material for: Mapping Thematic Trends and Analysing Hotspots Concerning the Use of Stem Cells for Cartilage Regeneration: A Bibliometric Analysis From 2010 to 2020
Source: Front Pharmacol. 2022 Jan 3;12:737939. doi: 10.3389/fphar.2021.737939 (PMC8762272; doi:10.3389/fphar.2021.737939)
Supplement: Supplementary file 1 [file Table1.docx]

**Table S1**

| **Id** | **Label** | **Cluster** | **Weight<Links>** | **Weight<Total link strength>** | **Weight<Occurrences>** | **Score<Avg. pub. year>** | **Score<Avg. citations>** | **Score<Avg. norm. citations>** |
| --- | --- | --- | --- | --- | --- | --- | --- | --- |
|  | adipose-derived mesenchymal stem cells | 1 | 57 | 84 | 9 | 2017.125 | 21.3333 | 1.1481 |
|  | adipose-derived stem cells | 1 | 75 | 195 | 25 | 2015.92 | 11 | 0.6974 |
|  | adipose-tissue | 1 | 131 | 477 | 57 | 2016.2857 | 27.3158 | 1.1689 |
|  | animal-models | 1 | 70 | 158 | 19 | 2017.4444 | 15.4211 | 0.7591 |
|  | arthritis | 1 | 74 | 148 | 18 | 2017.2222 | 13.6667 | 0.691 |
|  | articular cartilage | 1 | 109 | 337 | 40 | 2017.4444 | 21.425 | 0.9889 |
|  | articular-cartilage | 1 | 187 | 1995 | 238 | 2016.8448 | 24.0084 | 1.0571 |
|  | articular-cartilage defects | 1 | 84 | 214 | 22 | 2016.619 | 31.5455 | 0.8281 |
|  | autologous chondrocyte implantation | 1 | 136 | 763 | 88 | 2017 | 25.6932 | 1.2049 |
|  | autologous chondrocyte transplantation | 1 | 69 | 123 | 14 | 2014.7143 | 40.6429 | 1.1597 |
|  | bone marrow | 1 | 49 | 61 | 8 | 2015.5 | 46.25 | 1.3233 |
|  | bone marrow-derived mesenchymal stem cells | 1 | 49 | 70 | 9 | 2018.625 | 7.1111 | 2.0284 |
|  | bone-marrow | 1 | 178 | 1572 | 193 | 2016.8316 | 22.3679 | 1.0561 |
|  | cartilage defect | 1 | 70 | 141 | 17 | 2017.3125 | 7.0588 | 0.6091 |
|  | cartilage defects | 1 | 96 | 263 | 33 | 2015.9688 | 36.5758 | 1.349 |
|  | cartilage regeneration | 1 | 172 | 817 | 105 | 2017.18 | 18.6381 | 1.0819 |
|  | cartilage repair | 1 | 156 | 750 | 88 | 2017.8235 | 17.0909 | 1.0827 |
|  | cell therapy | 1 | 57 | 86 | 10 | 2016.4 | 23.8 | 0.754 |
|  | chondral defect | 1 | 54 | 106 | 10 | 2017.3333 | 8.9 | 0.4176 |
|  | chondral defects | 1 | 82 | 235 | 29 | 2015.2857 | 40.8966 | 1.0774 |
|  | chondrocyte implantation | 1 | 62 | 152 | 17 | 2015.9375 | 35.6471 | 1.2889 |
|  | chondrogenic progenitor cells | 1 | 57 | 103 | 12 | 2017.9 | 11.25 | 1.1723 |
|  | clinical-trial | 1 | 58 | 101 | 12 | 2018.25 | 14.4167 | 1.1367 |
|  | combination | 1 | 48 | 74 | 10 | 2016.8 | 8.4 | 0.3501 |
|  | defects | 1 | 150 | 847 | 104 | 2017.37 | 15.8654 | 1.0804 |
|  | disease | 1 | 38 | 56 | 8 | 2018.2857 | 13.25 | 1.864 |
|  | double-blind | 1 | 48 | 78 | 8 | 2018.75 | 11.125 | 1.6716 |
|  | efficacy | 1 | 40 | 67 | 8 | 2018.75 | 6.375 | 1.0072 |
|  | fibrin glue | 1 | 59 | 99 | 10 | 2016.8 | 18.9 | 0.9364 |
|  | follow-up | 1 | 87 | 202 | 22 | 2016.5909 | 23.6818 | 1.1432 |
|  | full-thickness defects | 1 | 86 | 226 | 26 | 2015.2 | 29.4615 | 1.1455 |
|  | growth-factor | 1 | 90 | 205 | 25 | 2016.24 | 34 | 1.1723 |
|  | high tibial osteotomy | 1 | 46 | 127 | 16 | 2018.4375 | 16.1875 | 2.0821 |
|  | hyaluronic-acid | 1 | 112 | 360 | 45 | 2017.9091 | 11.7556 | 1.142 |
|  | implantation | 1 | 114 | 374 | 42 | 2017.3171 | 21.2619 | 0.8652 |
|  | infrapatellar fat pad | 1 | 76 | 143 | 16 | 2016.3333 | 34.625 | 1.4944 |
|  | injection | 1 | 52 | 72 | 10 | 2017.3 | 24 | 1.3007 |
|  | injury | 1 | 46 | 95 | 13 | 2018 | 27.4615 | 1.5298 |
|  | intra-articular injection | 1 | 66 | 155 | 16 | 2017.125 | 63.3125 | 2.579 |
|  | intraarticular injection | 1 | 115 | 524 | 60 | 2017.5424 | 25.8833 | 1.5449 |
|  | joint | 1 | 57 | 127 | 16 | 2017.0667 | 32.125 | 1.7487 |
|  | knee | 1 | 154 | 1065 | 122 | 2016.8898 | 24.2951 | 1.0771 |
|  | knee osteoarthritis | 1 | 118 | 383 | 48 | 2017.9574 | 13.7083 | 0.9536 |
|  | knee-joint | 1 | 65 | 100 | 11 | 2017.1818 | 26.0909 | 1.4808 |
|  | lesions | 1 | 57 | 127 | 14 | 2017 | 17.6429 | 0.8584 |
|  | meniscus | 1 | 52 | 88 | 11 | 2016.8 | 25.1818 | 0.9586 |
|  | mesenchymal stem cell | 1 | 151 | 677 | 82 | 2016.6173 | 22.439 | 0.9654 |
|  | mesenchymal stem cells | 1 | 180 | 1479 | 177 | 2016.9195 | 21.1525 | 1.124 |
|  | mesenchymal stromal cells | 1 | 117 | 405 | 50 | 2016.625 | 18.28 | 1.025 |
|  | microfracture | 1 | 110 | 450 | 51 | 2016.44 | 22.5882 | 0.9882 |
|  | microfracture technique | 1 | 49 | 82 | 10 | 2017.6667 | 16.9 | 1.0008 |
|  | model | 1 | 89 | 250 | 32 | 2016.4062 | 31.2812 | 1.3176 |
|  | mscs | 1 | 70 | 150 | 21 | 2017.4 | 19 | 1.1401 |
|  | osteoarthritis | 1 | 193 | 2133 | 264 | 2017.593 | 19.5341 | 1.2344 |
|  | osteochondral defect | 1 | 97 | 256 | 30 | 2017.4138 | 12.3667 | 0.8426 |
|  | osteochondral defects | 1 | 106 | 307 | 35 | 2015.9118 | 41.4286 | 1.0614 |
|  | osteochondral lesions | 1 | 35 | 70 | 8 | 2016.5 | 72.75 | 2.6564 |
|  | outcomes | 1 | 42 | 57 | 8 | 2018.125 | 25.125 | 1.5351 |
|  | platelet-rich plasma | 1 | 122 | 529 | 66 | 2017.7656 | 16.1515 | 1.0508 |
|  | rabbit | 1 | 65 | 119 | 13 | 2016.1667 | 26.7692 | 0.8191 |
|  | rat | 1 | 39 | 64 | 8 | 2016.5 | 30.375 | 1.1772 |
|  | rat model | 1 | 41 | 61 | 9 | 2018.3333 | 9.1111 | 0.8044 |
|  | regeneration | 1 | 190 | 1813 | 222 | 2017.2279 | 21.5856 | 1.0202 |
|  | regenerative medicine | 1 | 143 | 582 | 71 | 2016.7857 | 20.5493 | 0.8282 |
|  | repair | 1 | 193 | 2194 | 273 | 2017.2462 | 21.3407 | 1.0199 |
|  | stem cell | 1 | 91 | 207 | 24 | 2017.9583 | 20 | 1.2915 |
|  | stem-cells | 1 | 121 | 366 | 49 | 2018.6809 | 7.5918 | 0.9928 |
|  | subchondral bone | 1 | 79 | 195 | 21 | 2017.55 | 12.1429 | 0.7771 |
|  | synovial-fluid | 1 | 59 | 99 | 12 | 2018.6 | 5.25 | 1.6291 |
|  | synovium | 1 | 71 | 169 | 19 | 2016.6842 | 12.7895 | 1.0838 |
|  | therapies | 1 | 40 | 58 | 8 | 2015.875 | 32.625 | 2.1875 |
|  | therapy | 1 | 132 | 635 | 83 | 2016.9756 | 33.988 | 1.6095 |
|  | transplantation | 1 | 148 | 772 | 92 | 2016.4494 | 23.4891 | 0.9989 |
|  | trial | 1 | 48 | 89 | 11 | 2016.9091 | 24.9091 | 1.3736 |
|  | acid | 2 | 64 | 115 | 15 | 2016.0667 | 14.6667 | 0.6241 |
|  | activation | 2 | 72 | 144 | 20 | 2016.35 | 16.5 | 0.8412 |
|  | adhesion | 2 | 56 | 89 | 13 | 2017.25 | 24.6154 | 0.7888 |
|  | angiogenesis | 2 | 45 | 60 | 9 | 2017.6667 | 13 | 0.9682 |
|  | apoptosis | 2 | 66 | 123 | 18 | 2018 | 14.3333 | 1.0848 |
|  | articular chondrocytes | 2 | 96 | 258 | 32 | 2016.9375 | 16.5625 | 0.9579 |
|  | articular-cartilage repair | 2 | 66 | 116 | 15 | 2016 | 23.2 | 0.8111 |
|  | bmp-2 | 2 | 53 | 77 | 9 | 2015.1111 | 28.1111 | 0.6526 |
|  | bone marrow mesenchymal stem cells | 2 | 70 | 120 | 14 | 2018 | 10.0714 | 0.9251 |
|  | bone regeneration | 2 | 71 | 139 | 22 | 2017.6667 | 14.4545 | 0.628 |
|  | bone-formation | 2 | 47 | 70 | 11 | 2016.9 | 25.4545 | 1.2061 |
|  | cartilage | 2 | 189 | 1606 | 196 | 2016.8677 | 22.25 | 0.9966 |
|  | cartilage formation | 2 | 35 | 55 | 8 | 2014.875 | 23.25 | 0.6133 |
|  | chondrocyte | 2 | 123 | 382 | 49 | 2016.8085 | 27.9184 | 1.228 |
|  | chondrocyte differentiation | 2 | 58 | 94 | 15 | 2016.2 | 38.6 | 1.563 |
|  | chondrocytes | 2 | 177 | 1478 | 186 | 2016.9444 | 15.2258 | 0.8488 |
|  | chondrogenesis | 2 | 192 | 1691 | 208 | 2016.8408 | 17.1971 | 0.7985 |
|  | co-culture | 2 | 74 | 161 | 17 | 2016.2353 | 22.2941 | 1.1866 |
|  | coculture | 2 | 72 | 160 | 19 | 2016.6842 | 10.0526 | 0.6707 |
|  | culture | 2 | 113 | 307 | 42 | 2016.6 | 15.2143 | 0.6722 |
|  | decellularized extracellular matrix | 2 | 48 | 68 | 8 | 2017.1429 | 11.875 | 0.8926 |
|  | differentiation | 2 | 189 | 2120 | 282 | 2017.1741 | 16.2447 | 0.832 |
|  | endochondral ossification | 2 | 64 | 125 | 17 | 2018 | 10.1765 | 0.8313 |
|  | endothelial growth-factor | 2 | 39 | 59 | 10 | 2016 | 23.5 | 0.7089 |
|  | exosomes | 2 | 65 | 175 | 22 | 2018.7 | 46.9091 | 3.3548 |
|  | expansion | 2 | 73 | 179 | 22 | 2017.1818 | 18.8636 | 0.9872 |
|  | expression | 2 | 171 | 990 | 139 | 2017.1471 | 15.3741 | 0.8172 |
|  | extracellular vesicles | 2 | 53 | 102 | 12 | 2018.9091 | 22.4167 | 1.6479 |
|  | fibronectin | 2 | 50 | 78 | 9 | 2017.625 | 9.8889 | 0.3935 |
|  | gene | 2 | 45 | 59 | 10 | 2015.6667 | 13.7 | 0.7119 |
|  | gene-expression | 2 | 137 | 568 | 75 | 2016.2192 | 19.6 | 0.7964 |
|  | generation | 2 | 72 | 167 | 25 | 2017.3043 | 16 | 0.6675 |
|  | growth | 2 | 107 | 318 | 44 | 2017.619 | 17.9318 | 0.8891 |
|  | growth-factors | 2 | 91 | 250 | 30 | 2016.5 | 18.0667 | 0.6727 |
|  | human articular chondrocytes | 2 | 114 | 410 | 47 | 2016.6596 | 30.9574 | 1.3881 |
|  | human mesenchymal stem cells | 2 | 67 | 124 | 15 | 2016.5333 | 28.7333 | 1.3041 |
|  | hypertrophy | 2 | 107 | 301 | 36 | 2017.3429 | 9.9167 | 0.9713 |
|  | hypoxia | 2 | 59 | 117 | 15 | 2016.8 | 15.5333 | 0.8523 |
|  | identification | 2 | 42 | 67 | 12 | 2017.4167 | 14.75 | 0.6186 |
|  | in-vitro | 2 | 174 | 1322 | 170 | 2016.3593 | 24.0882 | 0.9854 |
|  | induction | 2 | 95 | 243 | 30 | 2016.9 | 15.2 | 0.9159 |
|  | inflammation | 2 | 82 | 168 | 23 | 2018.5652 | 20.7826 | 2.2144 |
|  | inhibition | 2 | 46 | 66 | 8 | 2016.5714 | 20.5 | 0.7622 |
|  | marrow | 2 | 66 | 142 | 18 | 2016.2778 | 13.8889 | 0.6238 |
|  | mesenchymal progenitor cells | 2 | 56 | 79 | 9 | 2015.5556 | 13.6667 | 0.3668 |
|  | microenvironment | 2 | 45 | 70 | 8 | 2017.125 | 14 | 1.2075 |
|  | msc | 2 | 44 | 64 | 8 | 2016.4286 | 12.625 | 0.8236 |
|  | n-cadherin | 2 | 46 | 69 | 9 | 2016.75 | 23.3333 | 0.8755 |
|  | osteogenesis | 2 | 58 | 125 | 18 | 2016.8333 | 21.5556 | 0.8609 |
|  | osteogenic differentiation | 2 | 131 | 452 | 64 | 2016.9677 | 15.3281 | 0.9572 |
|  | oxygen-tension | 2 | 43 | 61 | 8 | 2017.75 | 18.25 | 1.2381 |
|  | phenotype | 2 | 70 | 127 | 16 | 2016 | 15 | 0.6438 |
|  | progenitor cells | 2 | 134 | 512 | 63 | 2016.1452 | 29.4286 | 0.8934 |
|  | proliferation | 2 | 151 | 759 | 94 | 2017.0753 | 18.9787 | 1.141 |
|  | promotes | 2 | 39 | 54 | 8 | 2018.25 | 2.875 | 0.5284 |
|  | protein | 2 | 66 | 120 | 18 | 2016.9412 | 11.7222 | 0.9059 |
|  | receptor | 2 | 41 | 61 | 8 | 2018.125 | 12.75 | 1.1551 |
|  | redifferentiation | 2 | 54 | 92 | 11 | 2017.0909 | 16.3636 | 0.6908 |
|  | rheumatoid-arthritis | 2 | 37 | 55 | 8 | 2016.125 | 25.75 | 0.6711 |
|  | sox9 | 2 | 91 | 210 | 29 | 2016.8929 | 18.8966 | 0.678 |
|  | spheroids | 2 | 52 | 79 | 9 | 2018 | 17 | 1.4302 |
|  | stimulation | 2 | 47 | 84 | 11 | 2017.5 | 5.5455 | 0.9299 |
|  | stromal cells | 2 | 190 | 1520 | 184 | 2016.6648 | 25.6739 | 1.152 |
|  | tgf-beta | 2 | 95 | 242 | 29 | 2017.4828 | 18.4138 | 1.1032 |
|  | tissue | 2 | 187 | 1522 | 191 | 2016.8 | 17.178 | 0.7473 |
|  | umbilical-cord | 2 | 50 | 83 | 9 | 2017 | 13.8889 | 0.517 |
|  | 3d printing | 3 | 43 | 67 | 9 | 2019.625 | 7.6667 | 1.5154 |
|  | alginate | 3 | 64 | 110 | 12 | 2016.0909 | 12.5 | 0.5831 |
|  | biomaterials | 3 | 103 | 283 | 33 | 2018.129 | 10.7273 | 0.7543 |
|  | bmscs | 3 | 36 | 59 | 8 | 2017.625 | 16.625 | 0.7667 |
|  | bone | 3 | 158 | 771 | 97 | 2017.7234 | 11.8247 | 0.7883 |
|  | bone morphogenetic protein-2 | 3 | 55 | 76 | 10 | 2015.4444 | 34.8 | 0.8058 |
|  | cartilage tissue | 3 | 44 | 54 | 9 | 2016.3333 | 12.3333 | 0.4501 |
|  | cartilage tissue engineering | 3 | 122 | 407 | 49 | 2016.5532 | 20.3469 | 1.2819 |
|  | chitosan | 3 | 82 | 200 | 23 | 2017.3333 | 25.6087 | 1.23 |
|  | chondrogenic differentiation | 3 | 186 | 1648 | 203 | 2016.8737 | 18.6601 | 0.9346 |
|  | chondroitin sulfate | 3 | 47 | 93 | 10 | 2018.1 | 5.1 | 0.4004 |
|  | collagen | 3 | 126 | 392 | 47 | 2017.0851 | 15.5106 | 0.7528 |
|  | composite | 3 | 53 | 72 | 10 | 2017.8 | 7.1 | 0.9803 |
|  | controlled-release | 3 | 65 | 103 | 13 | 2017.1538 | 12.5385 | 1.3118 |
|  | cross-linking | 3 | 48 | 81 | 10 | 2017.875 | 13.1 | 0.9393 |
|  | degradation | 3 | 76 | 159 | 21 | 2016.9048 | 13.3333 | 1.1046 |
|  | delivery | 3 | 94 | 239 | 32 | 2016.75 | 25.2812 | 1.303 |
|  | design | 3 | 53 | 114 | 16 | 2018.4667 | 10 | 0.8995 |
|  | drug-delivery | 3 | 47 | 74 | 11 | 2019 | 7.4545 | 1.4271 |
|  | engineered cartilage | 3 | 106 | 275 | 31 | 2016.5862 | 22.3226 | 0.7628 |
|  | extracellular matrix | 3 | 92 | 214 | 26 | 2016.5 | 22.8846 | 0.9718 |
|  | extracellular-matrix | 3 | 122 | 452 | 57 | 2017.0536 | 25.3158 | 1.232 |
|  | fabrication | 3 | 50 | 65 | 10 | 2018.875 | 5.4 | 0.4312 |
|  | gelatin | 3 | 67 | 134 | 16 | 2017.6429 | 8.3125 | 0.7793 |
|  | growth factors | 3 | 58 | 83 | 8 | 2014.125 | 79.625 | 1.416 |
|  | hyaluronic acid | 3 | 98 | 209 | 22 | 2017.3182 | 27.7727 | 1.3538 |
|  | hyaluronic-acid hydrogels | 3 | 49 | 94 | 13 | 2017.1538 | 48.2308 | 1.8097 |
|  | hydrogel | 3 | 117 | 341 | 45 | 2017.619 | 17.4222 | 0.7495 |
|  | hydrogels | 3 | 120 | 430 | 51 | 2018.0625 | 8.0588 | 0.7374 |
|  | in-vivo | 3 | 116 | 280 | 37 | 2016.3143 | 23.9189 | 1.0149 |
|  | kartogenin | 3 | 54 | 98 | 11 | 2018.9 | 5.7273 | 1.0932 |
|  | marrow stromal cells | 3 | 106 | 286 | 36 | 2015.3611 | 32.75 | 1.1641 |
|  | matrix | 3 | 145 | 541 | 61 | 2016.8644 | 19.2623 | 0.9909 |
|  | mechanical-properties | 3 | 83 | 188 | 26 | 2017.7083 | 13.7692 | 1.0323 |
|  | mesenchymal stem-cells | 3 | 169 | 1117 | 152 | 2017.9857 | 15.9868 | 0.7546 |
|  | microspheres | 3 | 61 | 114 | 12 | 2016.6364 | 17.5833 | 1.0476 |
|  | nanoparticles | 3 | 42 | 61 | 9 | 2018.625 | 4.8889 | 0.4524 |
|  | osteochondral | 3 | 51 | 84 | 9 | 2018.1429 | 30.6667 | 1.8712 |
|  | osteochondral regeneration | 3 | 54 | 92 | 11 | 2019.5 | 4.8182 | 1.135 |
|  | pore-size | 3 | 73 | 143 | 19 | 2018.0526 | 16.9474 | 1.005 |
|  | rabbit model | 3 | 52 | 84 | 9 | 2017.375 | 12.4444 | 0.4801 |
|  | reconstruction | 3 | 58 | 91 | 12 | 2016.75 | 17.25 | 0.8043 |
|  | scaffold | 3 | 141 | 662 | 84 | 2016.8734 | 14.4405 | 0.6717 |
|  | scaffolds | 3 | 151 | 626 | 76 | 2017.7286 | 13.5526 | 0.9075 |
|  | silk fibroin | 3 | 52 | 94 | 11 | 2018 | 16 | 0.8708 |
|  | stem cells | 3 | 136 | 529 | 63 | 2016.8136 | 20.254 | 0.7771 |
|  | strategies | 3 | 53 | 80 | 10 | 2018.8 | 5.8 | 1.1186 |
|  | surface | 3 | 50 | 72 | 8 | 2017.8571 | 10.25 | 1.9109 |
|  | system | 3 | 37 | 51 | 8 | 2018.25 | 25.75 | 1.6343 |
|  | tgf-beta-1 | 3 | 58 | 104 | 12 | 2016.5833 | 22.75 | 0.9284 |
|  | tissue engineering | 3 | 171 | 932 | 108 | 2016.9714 | 20.537 | 0.9206 |
|  | tissue regeneration | 3 | 98 | 230 | 29 | 2016.5862 | 29.1724 | 1.3309 |
|  | tissue-engineered cartilage | 3 | 78 | 145 | 19 | 2016.6111 | 22.7368 | 1.0301 |
|  | tissues | 3 | 45 | 66 | 10 | 2017.6 | 11.3 | 0.8206 |
|  | transforming growth-factor-beta-1 | 3 | 52 | 76 | 10 | 2015.1 | 29.7 | 0.9995 |
|  | vitro | 3 | 57 | 85 | 11 | 2017.3636 | 23.7273 | 1.7422 |
|  | vivo | 3 | 67 | 108 | 15 | 2015.3333 | 31.2 | 1.0131 |
|  | growth-factor-beta | 4 | 57 | 94 | 11 | 2017.0909 | 14.1818 | 0.806 |
|  | human adipose-tissue | 4 | 58 | 124 | 14 | 2016.2308 | 18.2857 | 0.752 |
|  | human bone-marrow | 4 | 87 | 203 | 24 | 2014.6667 | 40.9583 | 1.291 |
|  | in-vitro chondrogenesis | 4 | 114 | 451 | 55 | 2015.9815 | 34.9818 | 1.2999 |
|  | umbilical-cord blood | 4 | 70 | 152 | 16 | 2015.4667 | 21.75 | 1.3242 |
