# Supplementary figures and images for: Mapping Thematic Trends and Analysing Hotspots Concerning the Use of Stem Cells for Cartilage Regeneration: A Bibliometric Analysis From 2010 to 2020
Source: Front Pharmacol. 2022 Jan 3;12:737939. doi: 10.3389/fphar.2021.737939 (PMC8762272; doi:10.3389/fphar.2021.737939)

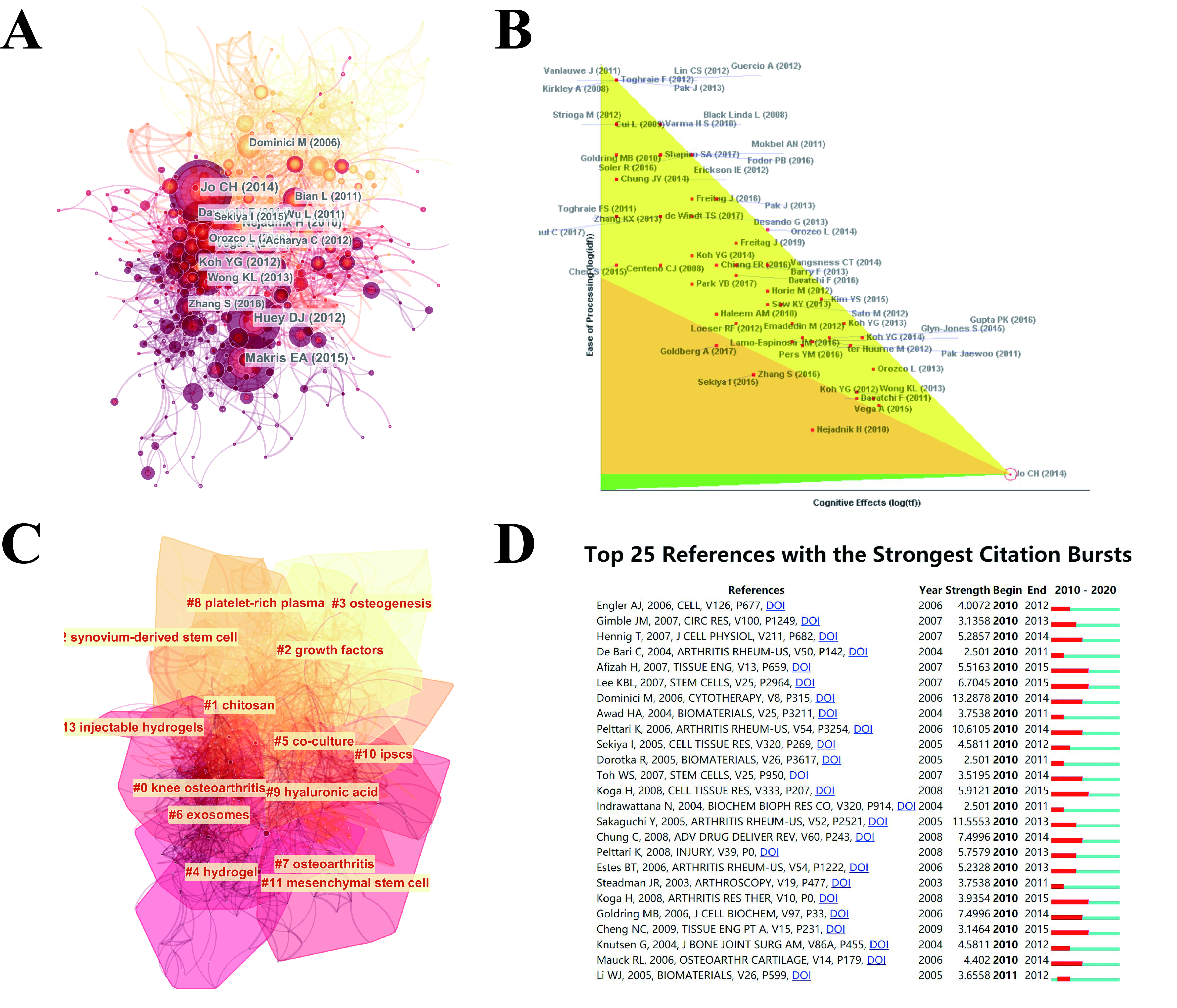

Supplement: Supplementary file 2 [file Image1.JPEG]

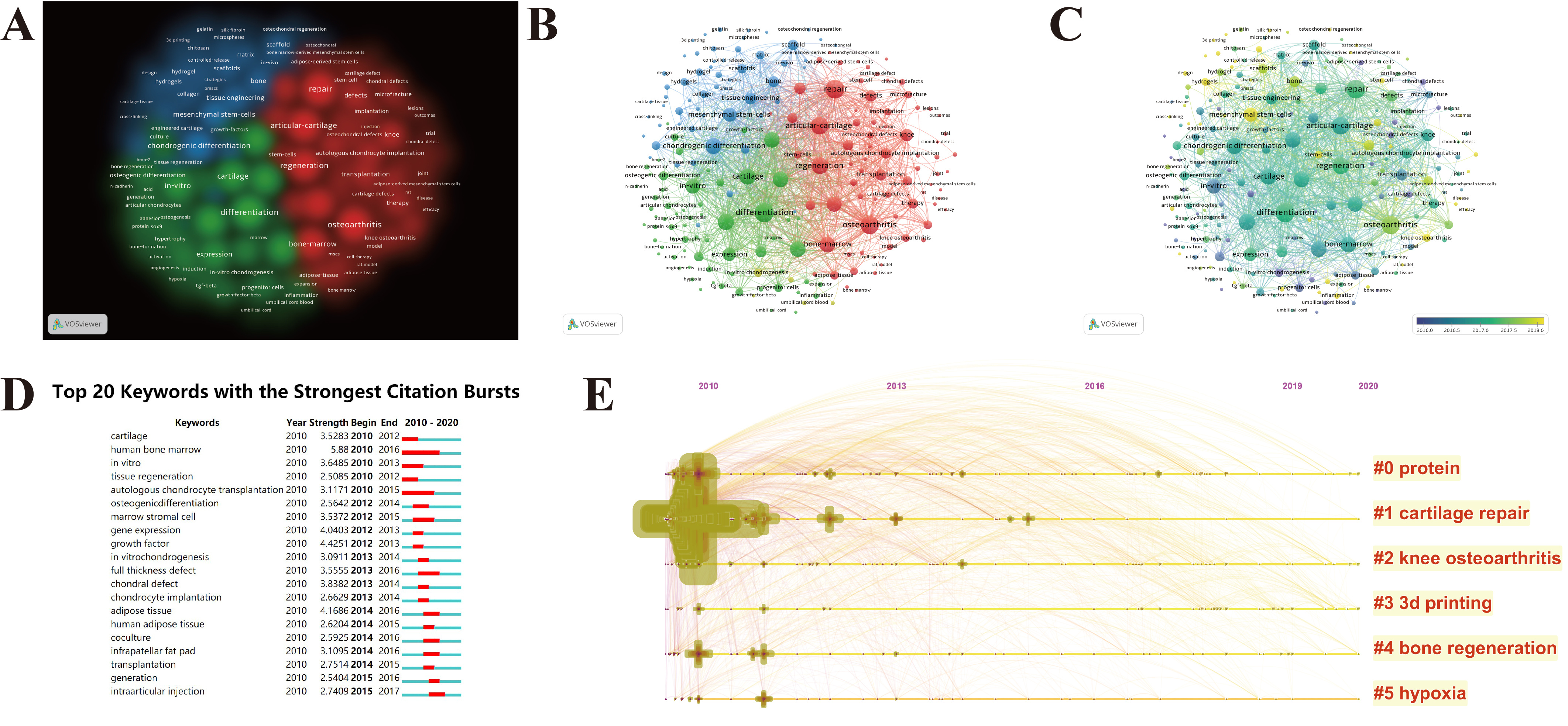

Supplement: Supplementary file 3 [file Image2.JPEG]
